# Policy and Practice Review: A First Guideline on the Use of Pharmacogenetics in Clinical Psychiatric Practice

**DOI:** 10.3389/fphar.2021.640032

**Published:** 2021-04-12

**Authors:** R. van Westrhenen, R. H. N. van Schaik, T. van Gelder, T. K. Birkenhager, P. R. Bakker, E. J. F. Houwink, P. M. Bet, W. J. G. Hoogendijk, M. J. M. van Weelden-Hulshof

**Affiliations:** ^1^ Outpatient Clinic Pharmacogenetics in Psychiatry, Parnassia Group Amsterdam, Amsterdam, Netherlands; ^2^ Maastricht Department of Psychiatry andNeuropsychology, Faculty of Health, Medicine and Life Sciences, Maastricht University, Maastricht, Netherlands; ^3^ Department of Clinical Chemistry, Erasmus Medical Center, Rotterdam, Netherlands; ^4^ Dept of Clinical Pharmacy and Toxicology, Leiden University Medical Center, Leiden, Netherlands; ^5^ Department of Psychiatry, Erasmus Medical Center, Rotterdam, Netherlands; ^6^ Institute for Mental Health, Arkin, Amsterdam, Netherlands; ^7^ Department of Public Health and Primary Care (PHEG), Leiden University Medical Centre, Leiden, Netherlands; ^8^ Department of Clinical Chemistry and Hospital Pharmacy, Amsterdam UMC Location VUMC, Amsterdam, Netherlands; ^9^ Pharmacy Ermel, Ermelo, Netherlands

**Keywords:** pharmacogenetics, clinical psychiatry, guideline, psychiatry, CYP = cytochrome P450, psychiatric treatment response, psychopharmaca, side effects

## Abstract

Effective pharmacologic treatments for psychiatric disorders are available, but their effect is limited due to patients’ genetic heterogeneity and low compliance-related to frequent adverse events. Only one third of patients respond to treatment and experience remission. Pharmacogenetics is a relatively young field which focusses on genetic analyses in the context of the metabolism and outcome of drug treatment. These genetic factors can, among other things, lead to differences in the activity of enzymes that metabolize drugs. Recently, a clinical guideline was authorized by the Dutch Clinical Psychiatric Association (NVvP) on the clinical use of pharmacogenetics in psychiatry. The main goal was to provide guidance, based on current evidence, on how to best use genotyping in clinical psychiatric practice. A systematic literature search was performed, and available publications were assessed using the GRADE methodology. General recommendations for psychiatric clinical practice were provided, and specific recommendations per medication were made available. This clinical guideline for caregivers prescribing psychotropic drugs is the product of a broad collaboration of professionals from different disciplines, making use of the information available at the Dutch Pharmacogenetics Working Group (DPWG) and the Clinical Pharmacogenetics Implementation Consortium (CPIC) so far. We summarize the relevant literature and all recommendations in this article. General recommendations are provided and also detailed recommendations per medication. In summary we advise to consider genotyping, when there are side effects or inefficacy for CYP2C19 and CYP2D6. When genotype information is available use this to select the right drug in the right dose for the right patient.

## Introduction

Effective treatments for mental disorders are available, but their effect is limited due to patients’ (genetic) heterogeneity and poor treatment compliance due to frequent adverse events. Only one-third of the patients respond to treatment and experience remission (SBU rapport Swedish Research Council, 2004; Walker et al., 2004). Studies (Porcelli et al., 2011; Altar et al., 2015) have shown that psychiatry relies on a trial-and-error approach that combines physicians’ experience with clinical indicators. Pharmacogenetic testing can help in this process by determining the person-specific genetic factors that may predict clinical response and side effects associated with genetic variants that impact drug-metabolizing enzymes, drug transporters or drug targets (Porcelli et al., 2011; Altar et al., 2015; van Westrhenen et al., 2020).

Pharmacogenetics is a discipline that investigates genetic factors that affect the absorption, metabolism, and transport of drugs, thereby affecting therapy outcome and pharmacogenetics might also include the study of genes involved in the mechanism of action of a drug. These genetic factors can, among other things, lead to differences in the activity of enzymes that metabolize drugs.

Genetic variants which are less than 1% present in the population are referred to as a mutation and more than 1% as genetic polymorphism. For several polymorphisms in genes encoding for metabolizing enzymes, the effect on the activity of the enzyme has been established. Besides genetic polymorphisms, also other (non-genetic) factors can influence enzyme activity, such as co-medication, smoking, diet and diseases (van Westrhenen et al., 2020). The genetic composition of the genes, referred to as the “genotype”, is translated into a predicted “phenotype”. Variants in cytochrome enzyme CYP2D6 are known to have the poor metabolizer (PM) phenotype in 5 to 10% of the population with a Northwestern European background (Zhou et al., 2017). These individuals have little or no CYP2D6 enzyme activity. They have an increased risk of side effects of medication that is metabolized by this particular enzyme, due to a slower degradation of certain drugs (for example with nortriptyline), and therefore higher blood drug concentrations. There also may be undertreatment if the drug has to be converted by CYP2D6 to the active metabolite, as is the case with some opioids (e.g., tramadol). Genetic polymorphisms influencing drug metabolism are common. Besides the poor metabolizer (PM) status, intermediate metabolizer status (IM: decreased enzyme activity) and ultrarapid metabolizer status (UM: increased enzyme activity) may predispose patients for an increased risk on both side effects and therapeutic ineffectiveness. For example, in the Caucasian population, 3% is CYP2C19 PM, 8% is CYP2D6 PM. Also, 17% is CYP2C19 IM and 30% CYP2D6 IM (Zhou et al., 2017).

When it comes to patients using psychotropic drugs, genotyping is at present only performed in clinical practice for genes encoding the cytochrome P450 enzymes (CYP), and thus focusses on the genetic variation in pharmacokinetic enzymes. These polymorphisms in the CYP genes are important as these CYP enzymes are of crucial importance for the metabolism of psychotropic drugs (van Westrhenen et al., 2020). This mainly concerns the genes encoding the CYP2C19 and CYP2D6 enzymes for which dosing recommendations have been defined and integrated into the electronic prescription and pharmacy systems in The Netherlands. In addition, detailed advice, including substantiation? is available to members of the Dutch Royal Pharmaceutical Society (KNMP) via a protected digital environment. Also, there is a freely accessible English PDF file (www.knmp.nl, search term pharmacogenetic guidelines). The Dutch medication advice as developed by the DPWG (Dutch Pharmacogenetic Working group) (Swen et al., 2011; Swen et al., 2018) has been largely adopted internationally by the CPIC (Clinical Pharmacogenetics Implementation Consortium, www.pharmgkb.org), and can be accessed freely at this website.

In current psychiatric clinical practice, patients are only genotyped when there is a medical need, i.e. when treatment has already started, and the patient is experiencing problems such as lack of efficacy or side effects (van Westrhenen et al., 2020). Therapeutic drug monitoring (TDM), in which drug concentrations in the blood are determined, is much more common when prescribing psychotropic drugs. For certain drugs, such as tricyclic antidepressants (TCA’s), lithium and clozapine, the application of TDM is of added value (Hiemke et al., 2018). For example, the risk of relapse is higher with low drug exposure, and proper dose adjustment is required to avoid higher doses and thus drug toxicity. However, TDM is not useful in the clinical use of all psychotropic drugs, especially for SSRIs and SNRIs where large interindividual differences in blood drug concentrations result in a lack of a reliable therapeutic window (www.tdm-monografie.org). One has to bear in mind that dosing advice for psychotropic drugs based on TDM is usually based on pharmacokinetic (PK) data, just like dose adjustments in renal impairments, without clinical randomized clinical trials supporting these recommendations.

Side effects may not only occur in patients with high drug doses and high plasma concentrations, but also at therapeutic doses in patients with a CYP PM status, and even in patients on a relatively low dose who have a CYP UM metabolizer status.

### Goal of Guideline Pharmacogenetics in Clinical Psychiatric Practice

A Dutch guideline “Pharmacogenetics in Clinical psychiatric practice clinical was drafted as assigned by the Dutch Psychiatric Association (NVvP) with funding from the Dutch Royal Medical Association (KNMG).” The main goal of this guideline was to provide guidance on how genotype could be used to contribute to best clinical psychiatric practice. The purpose was to address basic questions such as when to genotype, how to request genotyping, which genes to investigate and how to interpret the genotype results. Patients can also order genotyping via commercial companies, but this usually results in the outcome of more genes, for which not always sufficient evidence is present to be used in clinical practice. This guideline provides a description of dosing advice to be used in clinical psychiatric practice, based on current scientific evidence available. Because currently, insufficient data are available on the effects of genes involved in the pharmacodynamics of psychotropic drugs, these have been excluded from this paper but will be included in the updates to this guideline when more knowledge about the clinical applicability becomes available.

## Methods

The start of the project was instituting an official working group by the NVvP. Ten members were selected from official associations: the Dutch Hospital Pharmacy association (NVZA), Dutch Royal Pharmacy Association (KNMP), Dutch Association for Clinical Chemistry (NVKC), Dutch Internist Association (NIV), The Dutch College of General Practitioners (NHG), The Dutch Association for Clinical pharmacologists (NVKFB), the Dutch patient organization MIND and the Dutch Psychiatry Association NvvP. The initiative for this guideline, the writing of the funding application and the literature search were performed by the chair of the committee (RvW), after which discussions on the content of the papers was done by the entire group. Four main questions were formulated to be answered:

1. What is the effect of genotype vs. standard care on clinical outcome in psychiatric patients?
2. What is the preferred timing of genotyping?
3. Which enzymes to genotype?
4. Which recommendations: alternative drug or dose adjustment?

### Strategy for Literature Selection

Two independent researchers (RvW, AK) first searched existing websites and systematic reviews. Also the following electronic databases where searched (Dec 2017, updated lastly June 2019. Medline (via OVID), Embase (via Embase.com), the Cochrane Library (via Wiley), Web of Science, PsychINFO (via OVID) and Google Scholar for an extended list of search terms (see Supplementary Appendix 1).

The independent researchers selected studies based on predefined selection criteria: clinical studies, adult population, using validated questionnaires for the outcome (HDRS, CGI, MADRS, PANSS, BPRS, HAMD-A, HADS), pharmacogenes investigated (no proxy’s as e.g. metabolic ratio’s), English language, peer-reviewed publications, no chapters of conference abstracts, no case or family studies, depression, bipolar, schizophrenia, psychosis as diagnosed with DSM or ICD. Also, it was decided to focus on CYP genes as there are clear dose recommendations for these provided by DPWG and CPIC. In order to answer question 3 cross-sectional studies were also analyzed.

### Evaluation of Scientific Evidence

The power of the scientific evidence was rated according to the GRADE method (Grading Recommendations Assessment, Development and Evaluation, http://www.gradeworkinggroup.org/) as is common for evaluation of therapy or screening in the development of Dutch guidelines.

GRADE distinguishes four levels of quality of scientific evidence: high, moderate, low and very low. These levels refer to the certainty there is with regard to the conclusion (Brozek et al., 2009), see Table 1.

**TABLE 1:** GRADE levels of quality of scientific evidence.

GRADE	Definitie
High	•High certainty that true effect of treatment is close to estimated effect
	•Further research is very unlikely to change our confidence in the estimate of effect
	•Randomized trials without serious limitations well-performed observational studies with very large effects (or other qualifying factors),
Moderate	•Moderate certainty that true effect is close to estimated effect
	•Further research is likely to have an important impact on our confidence in the estimate of the effect and may change the estimate
	•Randomized trials with serious limitations, well-performed observational studies yielding large effects
Low	•Low certainty that true effect is close to estimated effect
	•Further research is very likely to have an important impact on our confidence in the estimate of effect and is likely to change
	•Randomized trials with very serious limitations, observational studies without special strengths or important limitations
Very low	•Any estimate of effect is very uncertain
	•Randomized trials with very serious limitations and inconsistent results
	•Observational studies with serious limitations, unsystematic clinical observations (e.g. case series or case reports.

### Formulation of Conclusions and Recommendations

For the relevant outcome measures, the scientific evidence was summarized in one or more literature conclusions, and the GRADE level was determined. Members of the guideline working group formulated an overall conclusion, and for recommendations, all relevant arguments were weighed by the working group.

### Considerations (from Evidence to Recommendations)

In order to formulate recommendations also other aspects were evaluated. Next to the quality of the scientific evidence, (clinical) expertise from working group members, preferences of patients as voiced by a patient member in the working group, as well as ethical considerations, costs and availability were also taken into account. These aspects were assessed by the working group.

### Official Authorization

The concept of guideline was submitted and reviewed three times by all involved (scientific and patient) official associations. All comments were collected and discussed within the working group, and the guideline was adjusted accordingly. This version of the guideline was finalized by the working group and authorized by the involved associations except the NvvP, that finalized a summary of the guideline in September 2020.

### Answer to Question 1. What Is the Effect of Genotyping Versus Standard Care on Clinical Outcome in Psychiatric Patients?

#### Summary of Literature

**FIGURE 1:**
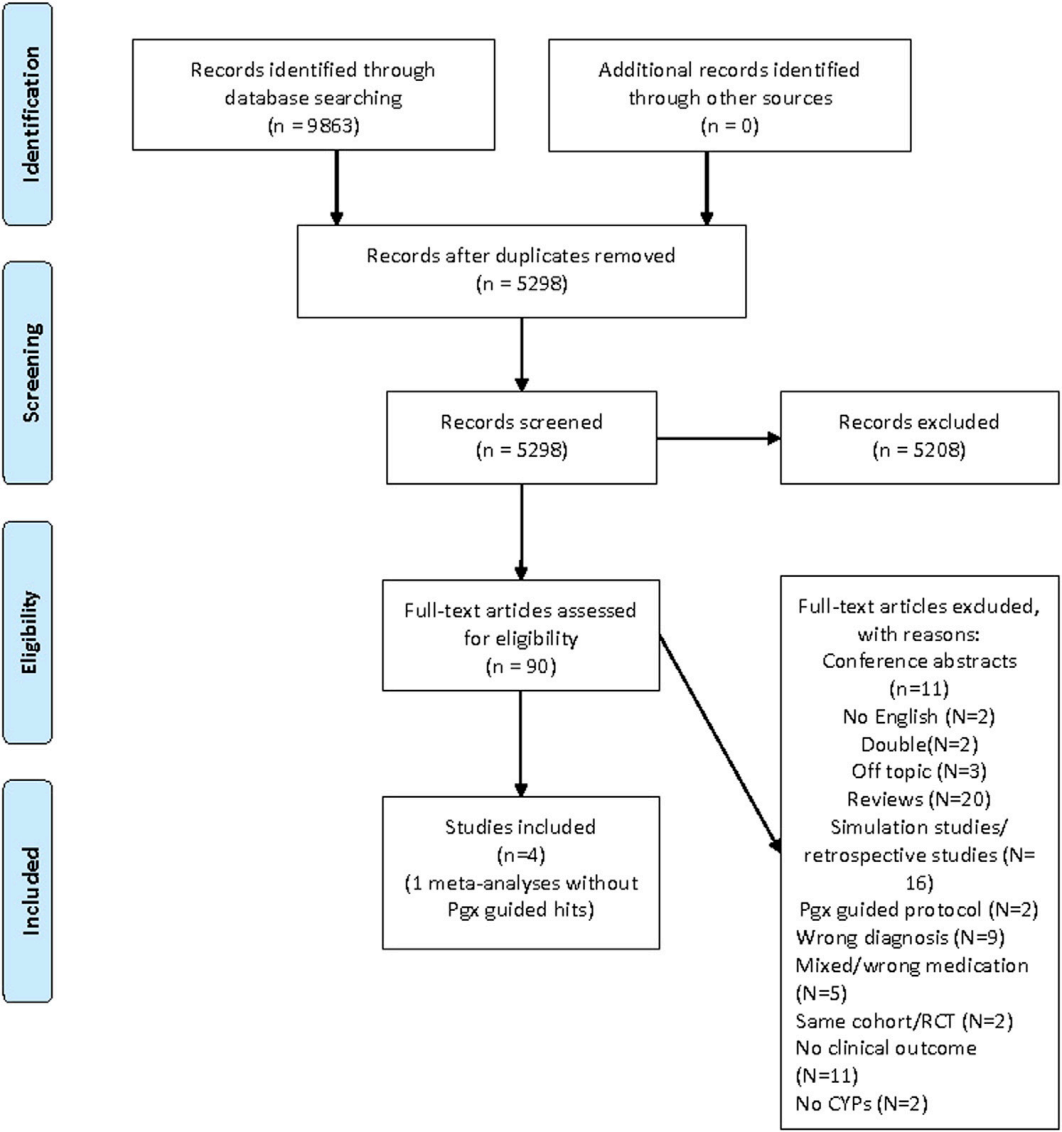
PRISMA 2009 flow diagram- genotyping in psychiatry.

The results of this literature study are summarized in Figure 1 PRISMA. In total, 8,863 hits were undoubled, leaving 5,298 references. All titles and abstracts were screened, and 5,208 references were excluded. In the end, 90 full-text articles were found and assessed. Only four prospective clinical studies were found (Figure 1. PRISMA Literature search Pharmacogenetics in Psychiatry), and based on these four clinical studies an official GRADE evaluation was performed. The four published clinical studies investigated the effect of genotyping compared to TAU in a total of n = 577 depressed patients.

Three studies made use of the Genesight test (Hall Falvin et al., 2012; Hall Falvin et al., 2013; Winner et al., 2013), and one of CNSDose (Singh 2015). Adult patients between 25 and 75 years with psychiatric diagnosis of depression and HAMD-17 scores over 14 were included. Patients used all kinds of different psychotropic agents and the studies were (at leats in part) sponsored by the suppliers of the pharmcogenetic kits. HAMD scores were evaluated after 10–12 weeks.

During the process of authorization of the guideline, three more recent studies were published. These were added to the already available clinical studies that were assessed for drafting this current publication and these seven prospective clinical studies are summarized in Table 2, entitled “Prospective Clinical Pharmacogenetic studies in Psychiatry.”

**Conclusion to Question 1**

**TABLE 2:** Prospective RCTs comparing PG guided vs non-guided pharmacotherapy, treatment as uual (TAU).

Genotyping method	Study design	Outcome
CYP1A2, CYP2D6, CYP2C19 (Genesight I)	Open label, prospective cohort: n = 25 genotyping guided vs n = 26 TAU (Hall-Flavin et al. (2012))	Genotyping leads to more reduction in depression scores
CYP1A2, CYP2D6 CYP2C19 (Genesight II)	Open label, prospective cohort: n = 114 genotyping guided vs n = 113 TAU (Hall-Flavin et al. (2013))	Genotyping leads to more reduction in depression symptoms
CYP1A2, 2C9, 2C19, 2D6, SCL6A4, 5HTR2A	RCT, double-blind: n = 26 guided vs n = 25 TAU (Singh (2015))	Genotyping results in higher response and remission rates
CYP2D6, CYP2C19, ABCB1, not otherwise specified	RCT, double-blind: n = 74 genotyping guided vs n = 74 TAU (Winner et al. (2013))	Genotyping 2.52 times more likely to remit
CYP2D6 and others, not specified		
(Neuro Pharmagen)	KT, double-blind: N = 155 guided vs n = 161 TAU (Perez et al. (2017))	Genotyping results in higher response rate and better tolerability
CYP1A2, 2C9, 2C19, 2D6, 3A4, 3A5, SCL6A4, COMT, HTR2A, MHFR (NeurolDgenetix)	RCT: n = 352 genotyping guided vs n = 333 TAU(Bradley et al. (2018))	Genotyping leads to higher response rates and remission rates in patients with depression or anxiety.
CYP1A2, 2C9, 2C19, 2B6, 2D6, HTR2A, SCL6A4	Prospective double-blind RCT: n = 1167 in total (Greden et al. (2019))	Genotyping leads to higher response and remission rates in depressed patients

### Answer to Question 2. What Is the Preferred Timing of Genotyping?

Literature Summary
No studies were found.
Results
No studies were found.
**Conclusion to Question 2**

### Answer to Question 3. Which Enzymes to Genotype?

**Conclusion to Question 3**

### Answer to Question 4. Which Recommendations: Alternative Drug or Dose Adjustment?

Which adjustments in pharmacotherapy are needed for patients that are using or starting psychotropic drugs and have a (CYP)genetic variant?

 **Conclusion to Question 4**

### Scope Guideline Pharmacogenetics in Psychiatry

This guideline provides recommendations on the implementation of CYP genotyping in patients with mood, anxiety and/or psychotic disorder, who are or will be taking antidepressants or antipsychotics. Since this description of the population is quite long, we use the shorter description ‘’psychiatric patients who (will) use antidepressants or antipsychotics”. In this guideline, we limit ourselves to variation in pharmacokinetic CYP enzymes. Currently, research on variation in, for example, transporters and receptors, such as, e.g. 5-HTTLPR (pharmacodynamic gene), is not advanced enough to give concrete recommendations in clinical psychiatric practice. Also, no dosing advice is provided for mood stabilizers and mutations in proteins that can lead to serious side effects as with carbamazepine and lamotrigine.

### Which Psychotropic Drug?

DPWG has drawn up medication advice for various groups of drugs, including antidepressants and antipsychotics, sometimes recommending adjusting the dosage or choosing a different drug. These agents are prescribed in patients with depressive disorders, anxiety disorders, psychotic disorders (e.g. schizophrenia or delirium), and many other conditions. In about one in three patients, the therapy fails due to side effects or inefficacy (SBU rapport Swedish Research Council, 2004; Walker et al., 2004).

• Medication advice is available for (www.kennisbank.knmp.nl):
• Antidepressants: amitriptyline, citalopram, clomipramine, doxepine, escitalopram, imipramine, nortriptyline, paroxetine, sertraline, venlafaxine
• Antipsychotics: aripiprazole, haloperidol, pimozide, zuclopenthixol
There is currently no substantiation for an advice to adjust therapy based on pharmacogenetics for (www.kennisbank.knmp.nl):
• Antidepressants: duloxetine, fluoxetine, fluvoxamine, mirtazapine, moclobemide
• Antipsychotics: clozapine, fluphenazine, flupentixol, olanzapine, quetiapine, risperidone, paliperidone
• What are the most important outcome measures that are relevant to the patient?
• The relevant outcome measures are the prevalence and severity of side effects and the clinical effect of the treatment.

### Intended Users of the Guideline

This guideline has been written for all prescribers and suppliers of antidepressants and antipsychotics, such as psychiatrists, general practitioners (GPs), neurologists, specialists in geriatric medicine, internists, clinical pharmacologists, and (hospital) pharmacists. The working group is aware that the recommendations included in this guideline are mainly related to the second or third line. However, primary care professionals can take note of the content of the guideline and the content can be included in the considerations of the general practitioner or pharmacist whether or not to genotyping. For GPs, the NHG has already published and updated (August 2020) a position on pharmacogenetics in general practice on its own initiative. For pharmacists there are of course the long-standing KNMP dosage recommendations. These recommendations are mostly based on pharmacokinetic studies. The purpose of this guideline Pharmacogenetics in psychiatry was to evaluate the clinically relevant scientific basis for the available dosage recommendations from the KNMP and the CPIC and to make recommendations that are applicable to the clinician practitioner in the treatment of psychiatric patients with psychopharmaca.Information specifically for patients is made available on several Dutch websites (www.apotheek.nl, and www.thuisarts.nl).

### Definitions and Concepts

What are the Main Definitions Used in this Guideline?

### Actionable Recommendations

#### Summary of the First Guideline Pharmacogenetics in Clinical Psychiatric Practice

An overview of the recommendations in this first Guideline Pharmacogenetics in clinical psychiatric practice is provided. Only pharmacokinetics and more specifically, the CYP-genotyping is discussed in psychiatric patients using antidepressants or antipsychotics. These recommendations should only be used in combination with other patient characteristics as comorbidity, comedication, medication history and the individual preference of the patient. Treatment should always be chosen based on mutual agreement between patients, significant others, doctors and other caregivers.

#### General recommendations for all psychopharmaca

When considering genotyping, inform the patient and involve the patient in order to achieve shared decision making with regard to genotype.
When genotype information is already available at the time of the prescription, use this information to select the right drug and the right dose for the right patient.
Consider genotyping, when there is an indication (side effects or inefficacy of CYP2D6 and CYP2C19. Preemptive genotyping is therefore not recommended yet for psychotropic drugs.
For some psychotropic drugs determination of CYP1A2, CYP2C9 and/or CYP3A4 can be of added value, but only determine those after consultation of a clinical pharmacologist or PharmD with pharmacogenetic expertise.
Ensure that available genotyping results are recorded in the (electronic) patient file and that this information is shared with medication prescribers (as the GP) as well as the pharmacy.
Consult a clinical pharmacologist or PharmD with specific knowledge in pharmacogenetics when in doubt before adjusting the drug dosage
For a psychotropic drug where no dose recommendation is provided (see Table 3 below): consider a switch to a different medicine form from a similar drug class, when there is a genetic variant in a CYP enzyme that is involved in the metabolization.

**TABLE 3:** Effect of genotyping on dosage, indicated as percentage of commonly prescribed dosage, as indicated by KNMP (01-01-2019) or Stingl et al. (2013).

Medicine	CYP2D6			CYP2C19			Relevant enzymes^#^
	PM	IM	UM	PM	IM	UM	
Amitriptyline	50%	60%	125%	70*	80%*	140%*	CYP2D6, CYP2C19
Aripiprazol	max 10 mg/dag	100%	100%	70%*	90%*	140%*	CYP2D6, CYP3A4
Citalopram	100%	100%	100%	50%	75%	100%	CYP2D6, CYP2C19, CYP3A4
Clomipramine	50%	70%	150%	65%*	75%*	130%*	CYP2D6, CYP2C19 (CYP3A4, CYP1A2)
Doxepine	40%	80%	200%	50%	90%	120%	CYP2D6 (CYP1A2, CYP3A4, 2C19)
Escitalopram	100%	100%	100%	50%	75%	150%	CYP2C19 (CYP3A4, CYP2D6)
Fluoxetine	100%	100%	100%				CYP2D6 (CYP2C19)
Haloperidol	50%	100%	NB				CYP2D6, CYP3A4
Imipramine	30%	70%	170%	70%	100%	100%	CYP2D6, CYP2C19
Nortriptyline	40%	60%	160%				CYP2D6, CYP2C19
Mirtazapine	100%	100%	100%				CYP2D6, CYP1A2, CYP3A4
Paroxetine	100%	100%	NB				CYP2D6
Pimozide	25%	60%	100%				CYP2D6, CYP3A4, CYP1A2
Risperidon	100%	100%	100%				CYP2D6 (CYP3A4)
Sertraline	100%	100%	100%	max 50 mg/dag	Max 100 mg	100%	CYP2C19 (CYP2D6, CYP2C9, CYP2B6, CYP3A4)
Venlafaxine	Not yet evaluated	Not yet evaluated	150%	40%*	90%*	125%*	CYP2D6, CYP3A4
Zuclopentixol	50%	75%	Not yet evaluated				

Of note: these percentages do not take into account co-medication, age, diet, renal function or comorbidity. Enzymes between brackets only have a minor contribution in the metabolization. Also, see www.kennisbank.knmp.nl for recent updates or consult a pharmacologist or PharmD. *.

**Table:** 

GRADE LOW	There is some evidence that pharmacogenetic-guided treatment with antidepressants results in a higher chance of remission of depression and better treatment response than standard care.
	Pharmacogenetic-guided treatment was formulated based on results of CYP1A2, CYP2C19 and CYP2D6. Pharmacodynamic genes were also determined but are outside the scope of this guideline.
	*Sources:* Hall-Flavin et al. (2012); Hall-Flavin et al. (2013); Singh (2015); Winner et al. (2013); Perez et al. (2017); Bradley et al. (2018); Greden et al. (2019)
-GRADE	No studies were found investigating the effect of pharmacogenetic-guided treatment compared to standard care in psychiatric patients using antipsychotics.
-GRADE	No studies were found investigating the effect of pharmacogenetic-guided treatment compared to standard care on side effects in psychiatric patients using antidepressants.

####  Specific recommendations per drug class

## Discussion

In 2005 the Dutch Pharmacogenetics Working group was formed. This working group formulates dosing advice based on pharmacokinetic metabolizer status. These dose recommendations have been available for all members of the Dutch Royal PharmD association at a secure website. This has so far hampered vast adoption of this knowledge as prescribers of medicines are usually not members. A guideline was drawn up by a working group consisting of psychiatrists (RvW, TB, WH), PharmD’s (MvW, PB), a GP (IH), a patient member (PU), an internist (TvG), clinical pharmacologists (RvW, TvG, PB), and a clinical chemist (RvS) to work together on translating the already available knowledge to clinical psychiatric practice. To assess the clinical evidence for the dose recommendations of the DPWG a literature study was performed collecting all clinical studies and evaluating these with the GRADE method. Although only four prospective clinical trials were found, that formed the basis for the recommendations in this publication, three more recent ones were published and added to this overview (Table 2). All studies yielded positive effects on clinical outcome when pharmacogenetic information was used to prescribe psychotropic drugs. Drawbacks of these studies were that they were sponsored by suppliers of the pharmacogenetic kits, different pharmacogenetic panels were used, not all were double-blind, they made use of different kinds of psychotropic drugs, and the algorithms that was used for dosing were not disclosed. That resulted in low GRADE evaluations of the Dutch NvvP working group and careful clinical recommendations.

This clinical guideline for caregivers prescribing psychotropic drugs is the product of a broad collaboration of professionals from different disciplines, making use of the information available at the Dutch Pharmacogenetics Working Group (DPWG) and the Clinical Pharmacogenetics Implementation Consortium (CPIC) so far. We summarized the relevant literature and all recommendations in this article. General recommendations are provided and also detailed recommendations per medication. In summary we advise to consider genotyping, when there are side effects or inefficacy for CYP2C19 and CYP2D6. When genotype information is available use this to select the right drug in the right dose for the right patient.

In the Netherlands, there is quite a unique situation because of the fact that 16 hospital pharmacies offer non-commercial pharmacogenetic testing. These tests are reimbursed by Dutch health insurance companies. The DPWG and the infrastructure in this small country make for good working alliances and cooperation between professionals from different centers in different areas and with worldwide collaborations. With this to our knowledge first clinical psychiatric guideline drawn up by clinicians working together with other professionals, a start can be made by implementing pharmacogenetics in clinical psychiatry at a larger scale. Through alignment with other initiatives as e.g. the CPIC and the recently started pan-European PSY-PGx research consortium that was set-up van Westrhenen, an international effort could be undertaken to establish worldwide clinical guidelines for implementation of pharmacogenetics in psychiatry.

There are still limitations that remain such as the lack of evidence for determining pharmacodynamic genes, the impact of rare genetic variants, possible roles of CYP genes in etiology of psychiatric diseases, and the ideal timing of genotyping. More studies will need to be undertaken and it seems The Netherlands could be the initiator of this because of the above infrastructural benefits. The potential benefit for psychiatric patients could be huge, which seems of extra importance in these uncertain (COVID) times where an increase in psychiatric disorders is expected.

**Table:** 

-GRADE	No prospective randomized clinical trials were found investigating genotyping before starting treatment with a psychopharmacon and comparing genotype adjusted dosing to standard care with regard to clinical outcome (clinical effect and/or side effects). These studies were not found for patients using antidepressants and neither for antipsychotics.

**Table:** 

GRADE LOW	There is low evidence for an association between using genotyping of CYP2C19 and CYP2D6 and clinical response to antidepressants in depressed patients.
	Bronnen: Hall-Flavin et al. (2012); Hall-Flavin et al. (2013); Winner et al. (2013); Singh (2015)

**Table:** 

-GRADE	No prospective clinical studies involving alternative drugs or dose adjustments were found that compared pharmacogenetic-guided strategies to standard prescribing of antidepressants or antipsychotics.

**Table:** 

Pharmacogenetics:	The study of the relationship between DNA sequence variations and drug response (EMEA/CHMP/ICH/437986/2006).
CYP enzymes:	Cytochrome P450 enzymes involved in the metabolism of a variety of drugs.
TDM:	Therapeutic drug monitoring: Measurement of medication levels in the blood, especially for drugs with a narrow therapeutic range, to avoid under- or overexposure. PM: Slow metabolizer (poor metabolizer), a person with absent or very low enzyme activity due to genetic variants for a specific metabolizing enzyme.
IM:	Intermediate metabolizer, person with low enzyme activity due to a genetic variant for a specific metabolizing enzyme.
EM/NM:	Normal metabolizer (extensive/normal metabolizer), a person with normal enzyme activity. A recent consensus article suggests changing this term to normal metabolizers (NM), and this has also been changed on the CPIC dosage recommendations (www.cpicpgx.org).
UM:	Ultrafast metabolizer (ultrarapid metabolizer), aperson with very high enzyme activity due to (multiple) genetic variants or copies of a specific metabolizing enzyme.

**Table:** 

Antidepressants Consider genotyping:
•In patients experiencing side effects or lack of efficacy after treatment with an adequate dose of an SSRI or SNRI. (see Table 2). Genotyping should be considered especially in patients that experienced side effects or inefficacy with multiple psychotropic drugs with a similar CYP metabolism.
•In patients experiencing side effects or unexplained high or low blood drug levels in patients using TCA (tricyclic antidepressants).
•When next to above, there are side effects and/or inefficacy with other (somatic) pharmaca with similar CYP metabolism.
Antipsychotics Consider genotyping:
•When patients experience side effects or lack of efficacy with antipsychotics other than clozapine, where medication advise is available (see Table 2). This should considered especially in patients that experienced side effects or inefficacy using other antipsychotics also with similar CYP metabolism.
•When next to above there are side effects and/or inefficacy with other (somatic) pharmaca with similar CYP metabolism.
Clozapine
When starting clozapine treatment use the blood level of the drug (TDM; therapeutic drug monitoring). Also, use TDM to optimize clozapine treatment. CYP1A2 genotyping is of limited value.
Lithium
Do not use genotyping for CYP enzymes for lithium treatment as lithium is cleared unchanged by the kidney and not by CYP enzymes.
Consider dose adjustment when starting the medication described in Table 2 for different phenotypes. Also, take into account possible interactions resulting from comedication or diet.

## References

[B1] Altar C. A. Carhart J. Allen J. D. (2015). Clinical utility of combinatorial pharmacogenomics-guided antidepressant therapy: evidence from three clinical studies. Mol. Neuropsychiatry. 1 (3), 145–155. 10.1159/000430915 27606312 PMC4996033

[B2] Bradley P. J. Shiekh M. Mehra V. Vrbicky K. Layle S. Olson M. C. (2018). Improved efficacy with targeted pharmacogenetic-guided treatment of patients with depression and anxiety: a randomized clinical trial demonstrating clinical utility. J. Psychiatr. Res. 96, 100–107. 10.1016/j.jpsychires.2017.09.024 28992526

[B3] Brozek J. L. Akl E. A. Alonso-Coello P. Jaeschke R. Williams J. D. Phillips B. (2009). GradingGrading quality of evidence and strength of recommendations in clinical practice guidelines. Part 1 of 3. An overview of the GRADE approach and grading quality of evidence about interventions. Allergy 64, 669–677. 10.1111/j.1398-9995.2009.01973.x 19210357

[B4] Greden J. F. Parikh S. V. Rothschild A. J. Thase M. E. Dunlop B. W. DeBattista C. (2019). Impact of pharmacogenomics in major depressive disorder in the GUIDED trial: a large, patient-and rater-blinded, randomized, controlled study. J. Psych Res. (111), 59–67. 10.1016/j.jpsychires.2019.01.003 30677646

[B5] Hall-Flavin D. K. Winner J. G. Allen J. D. Jordan J. J. Nesheim R. S (2012). Using a pharmacogenomic algorithm to guide the treatment of depression. Transl. Psychiatry. 2, e172. 10.1038/tp.2012.99 23047243 PMC3565829

[B6] Hall-Flavin D. K. Winner J. G. Allen J. D. Joseph M. C. Brian P. Karen A. S. (2013). Utility of integrated pharmacogenomic testing to support the treatment of major depressive disorder in a psychiatric outpatient setting. Pharmacogenet. Genomics. 23 (10), 535–548. 10.1097/FPC.0b013e3283649b9a 24018772

[B7] Hiemke C. Bergemann N. Clement H. W. Conca A. Deckert J. Domschke K. (2018). Consensus guidelines for therapeutic drug monitoring in neuropsychopharmacology: update 2017. Pharmacopsychiatry 51 (1-2), 9–62. 10.1055/s-0043-116492 28910830

[B8] Perez V. Salavert A. Espadaler J. Tuson M. Saiz-Ruiz J. Saez-Navarro C. (2017). Efficacy of prospective pharmacogenetic testing in the treatment of major depressive disorder: results of a randomized, double-blind clinical trial. BMC Psychiatry. 17 (1), 250–251. 10.1186/s12888-017-1412-1 28705252 PMC5513031

[B9] Porcelli S. Drago A. Fabbri C. Gibiino S. Calati R. Serretti A. (2011). Pharmacogenetics of antidepressant response. J. Psychiatry Neurosci. 36 (2), 87–113. 10.1503/jpn.100059 21172166 PMC3044192

[B10] SBU Rapport Swedish Research Council (2004). Treatment of depression, a systematic review. ISBN 91-87890-87-9, 91-87890-887, 91-87890-94-1

[B11] Singh A. B. (2015). Improved antidepressant remission in major depression via a pharmacokinetic pathway polygene pharmacogenetic report. Clin. Psychopharmacol. Neurosci. 13 (2), 150–156. 10.9758/cpn.2015.13.2.150 26243841 PMC4540033

[B18] Stingl J. Brockmoller J. Viviani R. (2013). Genetic variability of drug-metabolizing enzymes: the dual impact on psychiatric therapy and regulation of brain function. Mol. Psychiary. 18 (3), 273–287. 10.1038/mp.2012.42 22565785

[B12] Swen J. J. Nijenhuis M. de Boer A. Grandia L. Maitland-van der Zee A. H. Mulder H. (2011). Pharmacogenetics: from bench to byte--an update of guidelines. Clin. Pharmacol. Ther. 89 (5), 662–673. 10.1038/clpt.2011.34 21412232

[B13] Swen J. J. Wilting I. de Goede A. L. Grandia L. Mulder H. Touw D. J. (2008). Pharmacogenetics: from bench to byte. Clin. Pharmacol. Thererratum Clin. Pharmacol. Ther. 8384 (51), 781175–781177. 10.1038/sj.clpt.6100507 18253145

[B14] van Westrhenen R. Aitchison K. J. Ingelman-Sundberg M. Jukic M. M. (2020). Pharmacogenomics of antidepressants and antipsychotic treatment: how far have we got and where are we going? Front. Psychiatry. 11, 94. 10.3389/fpsyt.2020.00094 32226396 PMC7080976

[B15] Walker E. kestler L. Bollini A. Hochman K. M. (2004). Schizophrenia: etiology and course. Annu. Rev. Psychol. 55, 401–430. 10.1146/annurev.psych.55.090902.141950 14744221

[B16] Winner J. G. Carhart J. M. Altar C. A. (2013). A prospective, randomized, double-blind study assessing the clinical impact of integrated pharmacogenomic testing for major depressive disorder. Discov. Med. 16 (89), 219–227. 24229738

[B17] Zhou Y. Ingelman-Sundberg M. Lauschke V. M. (2017). Worldwide distribution of cytochrome P450 alleles: a meta-analysis of population-scale sequencing projects. Clin. Pharmacol. Ther. 102 (4), 688. 10.1002/cpt.690 28378927 PMC5600063

