# Supplementary material for: Policy and Practice Review: A First Guideline on the Use of Pharmacogenetics in Clinical Psychiatric Practice
Source: Front Pharmacol. 2021 Apr 12;12:640032. doi: 10.3389/fphar.2021.640032 (PMC8117336; doi:10.3389/fphar.2021.640032)
Supplement: Supplementary file 1 [file datasheet1.pdf]

Addendum 1 Listing of Search terms for the Literature Search

The following search terms were used: ('pharmacogenetics'/de OR ('genotype'/exp AND ('pharmacology'/de)) OR (('genotype'/de OR 'genetic polymorphism'/exp OR 'phenotype'/exp OR 'allele'/de OR gene/de OR 'genetic screening'/de OR 'genetic susceptibility'/de OR 'genetic variability'/de) AND ('cytochrome P450'/de OR 'cytochrome P450 3A4'/de OR 'cytochrome P450 2D6'/de OR 'cytochrome P450 2C9'/de OR 'cytochrome P450 2C19'/de OR 'cytochrome P450 1A2'/de)) OR (pharmacogenetic* OR psychopharmacogenetic* OR (genotyp* NEAR/3 (pharmac*)) OR ((genetic* OR polymorph* OR genotyp* OR phenotyp* OR DNA-variation* OR gene OR allele*) NEAR/6 (P450 OR P-450 OR CYP-3a4 OR CYP3a4 OR CYP-2d6 OR CYP2d6 OR CYP-2c9 OR CYP2c9 OR CYP-2c19 OR CYP2c19 OR CYP-1a2 OR CYP1a2)) OR ((poor* OR ultra-rapid* OR ultrarapid* OR intermediate*) NEAR/3 metaboliz*)):ab,ti) AND ('psychiatry'/exp/mj OR 'mental disease'/mj OR 'mental hospital'/mj OR 'mental patient'/mj OR 'mental health care'/mj OR 'depression'/exp/mj OR 'psychosis'/exp/mj OR 'anxiety disorder'/exp/mj OR 'mood disorder'/mj OR 'psychopharmacology'/mj OR 'psychopharmacotherapy'/mj OR 'psychotropic agent'/mj OR 'antidepressant agent'/mj OR 'neuroleptic agent'/mj OR 'atypical antipsychotic agent'/mj OR 'haloperidol'/mj OR 'risperidone'/mj OR 'aripiprazole'/mj OR 'clomipramine'/mj OR 'imipramine'/mj OR 'nortriptyline'/mj OR 'paroxetine'/mj OR 'sertraline'/mj OR 'duloxetine'/mj OR 'venlafaxine'/mj OR 'mirtazapine'/mj OR 'moclobemide'/mj OR 'doxepin'/mj OR 'neuroleptic malignant syndrome'/mj OR 'delirium'/mj OR 'autism'/exp/mj OR (psychiatr* OR neuropsychiatr* OR psychotropic* OR psychopharmacogenetic* OR ((mental OR anxiet* OR mood) NEAR/3 (disease* OR disorder* OR patient* OR hospital* OR healthcare OR health-care)) OR depression* OR psychosis* OR psychoses* OR psychotic* OR antipsychotic* OR psychopharmac* OR antidepress* OR anti-depress* OR schizophreni* OR bipolar* OR neuroleptic* OR delirium OR delusion* OR autis* OR (obsessi* NEAR/3 compulsi*) OR hallucinat* OR haldol OR haloperidol OR risperidon* OR aripiprazol* OR clomipramin* OR imipramin* OR nortriptylin* OR paroxetin* OR sertralin* OR duloxetin* OR mirtazapin* OR doxepin* OR moclobemi* OR venlafaxin*):ab,ti) AND ('observational study'/exp OR 'cohort analysis'/exp OR 'longitudinal study'/exp OR 'retrospective study'/exp OR 'prospective study'/exp OR 'epidemiological data'/de OR 'case control study'/de OR 'cross-sectional study'/de OR 'correlational study'/de OR 'population research'/de OR 'family study'/de OR 'major clinical study'/de OR 'multicenter study'/de OR 'comparative study'/de OR 'follow up'/de OR 'clinical study'/de OR 'clinical article'/de OR 'clinical trial'/exp OR 'randomization'/exp OR 'intervention study'/de OR 'open study'/de OR 'community trial'/de OR 'controlled study'/de OR 'review'/exp OR 'systematic review'/exp OR 'meta-analysis'/de OR (((observation* OR epidemiolog* OR famil* OR comparativ* OR communit*) NEAR/6 (stud* OR data

OR research)) OR cohort* OR longitudinal* OR retrospectiv* OR prospectiv* OR population* OR (national* NEAR/3 (stud* OR survey)) OR (health* NEAR/3 survey*) OR ((case OR cases OR match*) NEAR/3 control*) OR (cross NEXT/1 section*) OR correlation* OR multicenter* OR multi-center* OR follow-up* OR followup* OR clinical* OR trial OR random* OR review* OR meta-analy*):ab,ti

pharmacogenetics psychiatry.
